# Hepatic Steatosis in People With Chronic Hepatitis B in Africa: Prevalence, Correlates, and Clinical Significance

**DOI:** 10.1111/jvh.70241

**Published:** 2026-10-04

**Authors:** Freeman W. Chabala, Edford Sinkala, Alana T. Brennan, Amy Miskimon Goss, Lamin Bojang, Yusupha Samateh, Alexander J. Stockdale, Roger Sombie, Arouna Sessouma, Alice Nanelin Guingane, Juste Aristide Goungounga, Mohamed El‐Kassas, Eman Marwan, Ibrahima Diallo, Abdoul Sow, Edith Okeke, Attah Okwute, Hans Prozesky, Zahiera Ismail, Mark Sonderup, C. Wendy Spearman, Philippa C. Matthews, Judicaël Malick Tine, Maud Lemoine, Hailemichael Desalegn Mekonnen, Asgeir Johnannessen, Michael J. Vinikoor, Hepatitis B in Africa Collaborative Network (HEPSANET), Alice Guingane, Roger Sombie, Asgeir Johannessen, Edith Okeke, Mohamed El‐Kassas, Gilles Wandeler, Ibrahima Diallo, Joan Rugemalila, Wendy Spearman, Hans Prozesky, Alex Stockdale, Maud Lemoine, Babatunde Duduyemi, Mary Afihene, Edford Sinkala, Asgeir Johannessen, Roger Sombie, Alex Stockdale, Alice Guingane, Michael Vinikoor, Edith Okeke, Maud Lemoine, Philippa Matthews, Wendy Spearman, Adria Ramirez

**Affiliations:** ^1^ Levy Mwanawasa Medical University Lusaka Zambia; ^2^ Department of Medicine University of Zambia Lusaka Zambia; ^3^ Department of Global Health and Epidemiology Boston University Boston USA; ^4^ Health Economics and Epidemiology Research Office, Faculty of Health Sciences University of the Witwatersrand Johannesburg South Africa; ^5^ Department of Nutrition Sciences University of Alabama at Birmingham Birmingham USA; ^6^ The Medical Research Council Unit the Gambia London School of Hygiene & Tropical Medicine Fajara The Gambia; ^7^ Malawi Liverpool Wellcome Research Programme Blantyre Malawi; ^8^ Department of Clinical Infection, Microbiology and Immunology University of Liverpool Liverpool UK; ^9^ Service D'hépato‐Gastro‐Entérologie, Centre Hospitalier Universitaire Yalgado Ouédraogo Ouagadougou Burkina Faso; ^10^ Université Lédea Bernard Ouédraogo Ouahigouya Burkina Faso; ^11^ Service D'hépato‐Gastro‐Entérologie, Centre Hospitalier Universitaire Bogodogo Ouagadougou Burkina Faso; ^12^ Joseph Ki‐Zerbo University Ouagadougou Burkina Faso; ^13^ Université de Rennes EHESP, CNRS, INSERM, Arènes‐UMR 6051, RSMS‐U1309 Rennes France; ^14^ Endemic Medicine Department, Faculty of Medicine Capital University Cairo Egypt; ^15^ Hôpital Principale de Dakar Department of Immunology, Faculty of Medicine, Pharmacy, and Odontology Cheikh Anta Diop University of Dakar Dakar Senegal; ^16^ Department of Internal Medicine and Hepato‐Gastroenterology Hôpital Principal de Dakar Dakar Senegal; ^17^ University of Jos and Jos University Teaching Hospital Jos Nigeria; ^18^ Division of Infectious Diseases, Department of Medicine Tygerberg Hospital and Stellenbosch University Cape Town South Africa; ^19^ Division of Hepatology, Department of Medicine, Faculty of Health Sciences University of Cape Town Cape Town South Africa; ^20^ The Francis Crick Institute London UK; ^21^ University College London London UK; ^22^ Service de Maladies Infectieuses et Tropicales Centre Hospitalier National, Universitaire de Fann Dakar Senegal; ^23^ Division of Digestive Diseases, Department of Metabolism, Digestion and Reproduction Imperial College London London UK; ^24^ St. Paul's Hospital Millennium Medical College Addis Ababa Ethiopia; ^25^ Toronto Center for Liver Disease at University Health Network, Toronto General Hospital University of Toronto Toronto Canada; ^26^ Department of Infectious Diseases Vestfold Hospital Tønsberg Norway; ^27^ Institute of Clinical Medicine University of Oslo Oslo Norway; ^28^ Global Infectious Disease Research, Department of Medicine University of Alabama at Birmingham Birmingham USA; ^29^ Centre for Infectious Disease Research in Zambia Lusaka Zambia

**Keywords:** Africa, chronic HBV infection, HBV/HIV coinfection, liver fibrosis, steatosis

## Abstract

To address data gaps around metabolic liver disease in Africa, within the Hepatitis B in Africa Collaborative Network (HEPSANET), we evaluated participants with chronic hepatitis B virus infection (CHB) for steatotic liver disease (SLD) using transient elastography with controlled attenuation parameter (CAP). Using multivariable logistic regression, we identified sociodemographic, anthropometric, and clinical associations with SLD (CAP ≥ 248 dB/m), and evaluated whether SLD severity was independently associated with fibrosis. Short‐term impacts of SLD on mortality were also assessed with Cox proportional hazards analysis. We also evaluated the diagnostic performance of B‐mode ultrasound for SLD, with CAP as reference. Across 6 countries and 1601 participants (age range, 12–80 years; 39.5% female; 4% with diabetes; 11% unhealthy alcohol use; median body mass index, 24.4), 421 (26.3%) had SLD; 219 (13.7%) had severe steatosis (CAP ≥ 280 dB/m). Older age, male sex, overweight and obesity, and HIV‐negative status were associated with SLD. After adjusting for age, sex, HIV status, HBV treatment, and country, severe steatosis, but not mild and moderate forms (i.e., 248–279 dB/m), was associated with a 8.4% (95%, 1.9%–14.9%) increased probability of fibrosis. Over limited follow‐up (median 2.69 years) and small number of deaths, SLD was not associated with all‐cause or liver‐related mortality. B‐mode ultrasound had low sensitivity for SLD, overall or when severe, but specificity was 95%. In conclusion, SLD was common in people with CHB in Africa and its severe form was associated with liver fibrosis. These data support screening for metabolic comorbidities as part of HBV care and treatment in Africa.

## 1 Introduction

Steatotic liver disease (SLD) is highly prevalent worldwide and is mainly attributed to metabolic‐dysfunction associated liver disease (MASLD) or alcohol‐associated liver disease (ALD). Worldwide, 32% of adults are estimated to have MASLD and this prevalence has significant increased over the past two decades [1]. In the absence of unhealthy alcohol use, SLD is a criterion for MASLD when accompanied by metabolic factors such as obesity, essential hypertension, dyslipidemia, or Type 2 diabetes melitus [2]. SLD has become a major cause of chronic liver disease globally and although it is a reversible liver condition, it can induce liver inflammation and fibrosis with a risk of cirrhosis and hepatocellular carcinoma (HCC). Currently, most data on SLD come from high‐income countries; however, there is a paucity of evidence on its epidemiology in low and middle‐income countries (LMICs), including in the African region, where chronic liver disease is a major cause of mortality [3].

In Africa, SLD and its promoting factors intersect with chronic hepatitis B (CHB), the leading cause of HCC and liver‐related death in the region [4]. An estimated 64.7 million people are living with CHB in Africa and the incidence of cardiometabolic conditions is rapidly rising due to an ageing population and increasing urbanization, economic transition toward sedentary lifestyles, and adoption of diets that include more ultra processed and high‐sugar foods, creating an environment that can promote SLD‐HBV comorbidity [5]. Africa lags in HBV elimination and within clinics treating HBV, there is limited appreciation for the need to screen for SLD, as is done for alcohol use disorders. The clinical significance of SLD in the absence of other cardiometabolic risk factors is also unclear with conflicting evidence on its impact on clinical outcomes. To call attention to HBV‐SLD comorbidity, in their 2024 update, the World Health Organization (WHO) guidelines on the management of CHB, which are often adopted for use in African countries, called for universal antiviral therapy for CHB in the presence of metabolic comorbidities, regardless of other HBV/liver‐related parameters, although evidence to support this recommendation was noted to be low and conflicting [6]. By contrast, the European Association for the Study of the Liver (EASL) Hepatitis B guidelines have not recommended treatment on the basis of MASLD, highlighting the conflicting evidence from cohort studies on its association with clinical outcomes [7].

This study set out to describe, among people with CHB, the prevalence, correlates, and clinical significance of comorbid SLD, based on transient elastography with controlled attenuation parameter (CAP). We analysed data from the Hepatitis B in Africa Collaborative Network (HEPSANET), which was designed to answer major HBV natural history and treatment questions for the region and currently includes data from many leading HBV research programs and clinical cohorts across multiple regions [8]. We hypothesized that SLD would be more common with obesity and with HIV infection, an HBV coinfection that is most common in Africa and associated with a higher risk of metabolic disorders [9]. We also hypothesized that SLD would be associated with liver fibrosis. Because TE with CAP is rarely available in the Africa region, we also explored the diagnostic performance of B‐mode ultrasound, using CAP as a reference test.

## 2 Materials and Methods

### 2.1 Study Design and Participants

HEPSANET manages a harmonized dataset of people with CHB (based on surface antigen positivity) from 15 sites in Africa [8]. Sites provide longitudinal HBV care and treatment and were selected to be in the network based on having research interest in HBV, having ethical approval to collect and use data for research, and having sufficient site data quality and quantity to contribute to peer‐reviewed publications. HIV status is routinely determined before/at enrollment via HIV‐1/2 antibody assays. Some sites manage and follow‐up people with HBV/HIV coinfection, whereas others refer patients with coinfection to other specialist care. HEPSANET includes sites that have existing prospective longitudinal cohort studies and sites that collect routine clinical data retrospectively. HBV treatment is per national guidelines in each country, which are often based on adapted versions of the WHO [6] or European Association for the Study of the Liver (EASL) guidelines [7].

HEPSANET sites often have the most advanced HBV clinical infrastructure in the region, including routine access to HBV DNA and serological assays, ultrasound, CT/MRI, and 13 of 15 sites use vibrational controlled transient elastography (TE) for non‐invasive assessment of liver fibrosis and cirrhosis, but access to diagnostic tests is heterogeneous between sites. TE devices at 8 HEPSANET sites also measure controlled attenuation parameter (CAP), a quantitative proxy measure of hepatic steatosis, which is considered a more sensitive screening tool than ultrasound [10].

Participants were included in this analysis if they were 12 years or older, enrolled in HBV care/research at one of the HEPSANET sites where CAP was available, and had at least one valid CAP score, defined as a median score of 100–400 dB/m based on 10 valid measurements, and success rate ≥ 60%. We excluded participants with a history of hepatitis C virus (HCV) or hepatitis delta virus (HDV) coinfections, based on anti‐HCV or anti‐HDV serology.

### 2.2 Variables

We analysed variables at the time of the first CAP measure for each participant. Our outcomes of interest were liver steatosis, fibrosis, and liver‐related mortality. Steatosis was primarily defined using TE CAP measure. We categorized steatosis as none (S0), based on median CAP (dB/m) < 248 dB/m; mild (S1) at 248–267, moderate (S2) at 268–279 dB/m, and severe (S3) at ≥ 280 dB/m [11]. We dichotomized the CAP score to describe any degree of steatosis (CAP ≥ 248 dB/m) and for severe steatosis (CAP ≥ 280 dB/m). We also evaluated B‐mode abdominal ultrasound, which is the lower‐cost and more widely available alternative for the diagnosis of steatosis. B‐mode ultrasound reports in HEPSANET included the presence or absence of steatosis. We categorized median liver stiffness as none‐minimal at < 7.0 kPa, significant‐advanced at 7.0–12.5 kPa, and cirrhosis at > 12.5 kPa as per WHO guidelines [6]. Other variables included demographic factors, including age, sex, education, and marital status, year of enrollment, body mass index, history of diabetes, history of unhealthy alcohol use, and the use of HBV antiviral therapy. HBV antiviral therapy was considered a proxy for higher HBV DNA, elevated ALT, and HBV e antigen positivity. Except for diabetes, we did not document other metabolic factors in our database; therefore, we were unable to define MASLD according to international criteria. We also described liver‐related mortality among those evaluated for SLD stratified by their steatosis severity categorization. For deaths, a mortality committee comprised of HEPSANET physicians systematically reviewed all available medical records to determine attribution to liver cancer and/or a complication of cirrhosis.

### 2.3 Statistical Methods

Within sites that had CAP available, we compared the sociodemographic and clinical characteristics among participants with and without CAP measures to discern bias in access to the test. When CAP interquartile ranges (IQR) were available in the dataset, we also calculated the IQR/median ratio, and we ran sensitivity analyses with exclusion of median CAP scores that were less precise based on an IQR/median ratio > 0.3. We used multivariable binomial logistic regression to evaluate patient characteristics associated with any grade of steatosis. Possible correlates were variables that were non‐missing for > 80% of analysed participants and were considered plausible factors associated with steatosis based on the extant literature. We considered sociodemographic factors, BMI, diabetes, history of unhealthy alcohol use, HIV status, use of HBV antiviral therapy, and country. We also evaluated associations of grades of steatosis (versus none) with significant fibrosis, after adjustment for age, sex, HIV status, and country. We estimated the average marginal effects to express associations as absolute differences in the predicted probability of fibrosis across steatosis grades.

We calculated person‐time of follow‐up from the date of CAP measurement to death, transfer or exit from the clinic/cohort, loss to follow‐up, or administrative censoring, whichever occurred first. We explored the association between steatosis grade (none [reference], mild, moderate, or severe) and all‐cause and liver‐related mortality using Cox proportional hazards models, with results expressed as hazard ratios (HRs) and 95% confidence intervals (CIs). Confounders—age, sex, HIV status, HBV treatment status, and country—were identified a priori using a directed acyclic graph (Figure S1). The proportional hazards assumption was assessed using Schoenfeld residual tests, and log‐linearity of continuous covariates was evaluated using Martingale residual plots.

Finally, we evaluated the diagnostic performance of B‐mode ultrasound to diagnose steatosis, using CAP as a reference standard. B‐mode ultrasounds were included if they were done within 6 months of CAP and reported the presence or absence of steatosis, based on increased hepatic echogenicity compared to the adjacent right renal cortex. All analyses were conducted in Stata version 19.5 (StataCorp, College Station, TX), with a two‐sided significance threshold of α = 0.05. Study sponsors had played no role in the design or implementation of the analysis or in the manuscript development or decision to publish it.

## 3 Results

Within HEPSANET, 8 of 15 sites, representing 6 countries in Africa, had the availability of CAP measurement. These sites contributed 5591 patients and of them, 1601 (28.6%) had a CAP measurement and were eligible for inclusion in this analysis (Figure 1). At sites where TE with CAP was available, compared to counterparts excluded for lack of CAP data, participants included in the analysis were older, were overweight/obesity, had HIV coinfection, and enrolled since 2010 (all *p* < 0.05; Table S1).

**FIGURE 1:**
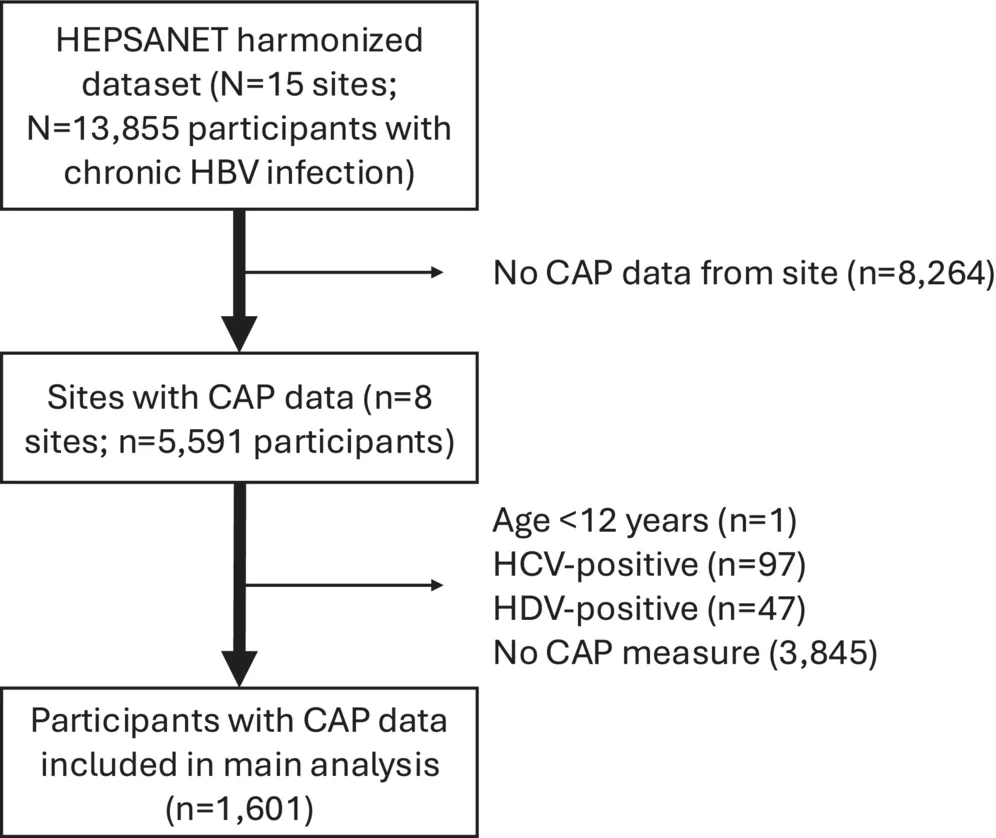
Flow diagram of study population selection. Boxes indicate the number of participants excluded at each step and the corresponding reason for exclusion.

Within the analysis group, the median age was 36.2 years (IQR, 29.9–44.7), 633/1601 (39.5%) were women, and 347/1601 (21.7%) had HBV/HIV coinfection. In addition, 216/1343 (16.1%) had obesity, 58/1420 (4.1%) had a history of diabetes, and 162 (11.2%) had a history of unhealthy alcohol use. Notably, 127 (9.5%) participants were underweight (i.e., BMI < 18.5). At their CAP measure, all participants with HBV/HIV were taking antiviral therapy, for a median of 6.3 years (IQR, 3.9–8.7). In HBV alone, 315 (25.1%) participants were on antiviral therapy at the time of CAP measure, for a median of 4.5 years (IQR, 1.4–7.5; Table 1).

**TABLE 1:** Characteristics of adults with chronic HBV infection evaluated for comorbid hepatic steatosis in HEPSANET.

	Overall (*N* = 1601)	No steatosis (*N* = 1180)	Steatosis (*N* = 421)
Age, median (IQR) years	36.2 (29.9–44.7)	34.5 (29.0–43.1)	40.5 (34.1–48.0)
Age, years			
12–29	403 (25.17)	350 (29.66)	53 (12.59)
30–39	575 (35.92)	429 (36.36)	146 (34.68)
40–49	380 (23.74)	252 (21.36)	128 (30.40)
50+	243 (15.18)	149 (12.63)	94 (22.33)
Sex			
Female	633 (39.54)	487 (41.27)	146 (34.68)
Male	968 (60.46)	693 (58.73)	275 (65.32)
Marital status			
Single	408 (28.20)	333 (31.56)	75 (19.13)
Married/cohabiting	969 (66.97)	664 (62.94)	305 (77.81)
Divorced/widowed	70 (04.84)	58 (05.50)	12 (03.06)
Missing	154	125	29
Education			
None/primary	273 (22.73)	224 (25.51)	49 (15.17)
Secondary	365 (30.39)	252 (28.70)	113 (34.98)
Tertiary and above	563 (46.88)	402 (45.79)	161 (49.85)
Missing	400	302	98
Body mass index			
< 18.5	127 (09.46)	121 (12.27)	6 (01.69)
18.5–24.9	607 (45.23)	516 (52.33)	91 (25.56)
25–29.9	392 (29.21)	244 (24.75)	148 (41.57)
> = 30	216 (16.10)	105 (10.65)	111 (31.18)
Missing	259	194	65
Diabetes history			
No	1361 (95.91)	995 (97.45)	366 (91.96)
Yes	58 (04.09)	26 (02.55)	32 (08.04)
Missing	182	159	23
HIV status			
Negative	1254 (78.33)	874 (74.07)	380 (90.26)
Positive	347 (21.67)	306 (25.93)	41 (09.74)
HBV DNA level (IU/ml)			
< = 25	474 (35.94)	357 (36.92)	117 (33.24)
26–2000	488 (37.00)	350 (36.19)	138 (39.20)
> 2000	357 (27.07)	260 (26.89)	97 (27.56)
Missing	282	213	69
HBeAg status			
Negative	621 (83.36)	437 (80.78)	184 (90.20)
Positive	124 (16.64)	104 (19.22)	20 (09.80)
Missing	856	639	217
Treatment status			
Not on antivirals	940 (58.71)	647 (54.83)	293 (69.60)
On antivirals	661 (41.29)	533 (45.17)	128 (30.40)
Country			
Burkina Faso	706 (44.10)	498 (42.20)	208 (49.41)
Egypt	102 (06.37)	59 (05.00)	43 (10.21)
Nigeria	40 (02.50)	28 (02.37)	12 (02.85)
Senegal	164 (10.24)	135 (11.44)	29 (06.89)
South Africa	459 (28.67)	343 (29.07)	116 (27.55)
Zambia	130 (08.12)	117 (09.92)	13 (03.09)
Unhealthy alcohol use			
No	1289 (88.84)	939 (87.68)	350 (92.11)
Yes	162 (11.16)	132 (12.32)	30 (07.89)
Missing	150	109	41

The overall median CAP value was 219 dB/m (IQR, 188–250). 421 (26.8%) participants had any degree of hepatic steatosis including 202 (12.6%) with mild–moderate (i.e., S1‐S2) and 219 (13.7%) with severe (i.e., S3) steatosis. In multivariable analysis associations with any degree of steatosis was associated with older age, male sex, overweight and obesity, and HIV‐negative status (Table 2). For example, compared to those with normal BMI, participants with overweight (AOR, 4.08; 95% CI, 2.70–6.17) and obesity (AOR, 6.34; 95% CI, 3.83–10.52) were associated with greater odds of steatosis. People with HIV coinfection had 74% reduced odds of steatosis (AOR, 0.26; 95% CI, 0.10–0.71; Table 2).

**TABLE 2:** Correlates of evaluation for steatosis with TE CAP.

	Crude OR (95% CI)	Adjusted OR (95% CI)
Age		
12–29	Reference	Reference
30–39	2.25 (1.59–3.17)	2.27 (1.33–3.86)
40–49	3.35 (2.34–4.80)	3.28 (1.82–5.93)
50+	4.17 (2.83–6.14)	4.00 (2.01–7.95)
Sex		
Female	Reference	Reference
Male	1.32 (1.05–1.67)	1.72 (1.17–2.52)
Marital status		
Single	Reference	Reference
Married/cohabiting	2.04 (1.53–2.71)	0.95 (0.60–1.51)
Divorced/widowed	0.92 (0.47–1.80)	0.68 (0.21–2.23)
Education		
None/primary	Reference	Reference
Secondary	2.05 (1.40–3.00)	1.75 (0.99–3.10)
Tertiary and above	1.83 (1.28–2.62)	1.47 (0.86–2.50)
Body mass index		
< 18.5	0.28 (0.12–0.66)	0.15 (0.02–1.09)
18.5–24.9	Reference	Reference
25–29.9	3.44 (2.54–4.65)	4.08 (2.70–6.17)
> = 30	5.99 (4.23–8.49)	6.34 (3.83–10.52)
Diabetes history		
No	Reference	Reference
Yes	3.61 (2.12–6.14)	1.97 (0.77–5.03)
HIV status		
Negative	Reference	Reference
Positive	0.31 (0.22–0.44)	0.26 (0.10–0.71)
Treatment status		
Not on antivirals	Reference	Reference
On antivirals	0.53 (0.42–0.67)	0.78 (0.50–1.20)
Unhealthy alcohol use		
No	Reference	Reference
Yes	0.61 (0.40–0.92)	0.50 (0.20–1.24)
Country		
Burkina Faso	Reference	Reference
Egypt	1.74 (1.14–2.67)	1.43 (0.70–2.91)
Nigeria	1.03 (0.51–2.06)	1.92 (0.24–15.16)
Senegal	0.51 (0.33–0.79)	0.38 (0.19–0.76)
South Africa	0.81 (0.62–1.06)	0.77 (0.45–1.30)
Zambia	0.27 (0.15–0.48)	^a^

Abbreviations: CHB, chronic hepatitis B virus infection; CI, confidence interval; HEPSANET, Hepatitis B in Africa collaborative network; HIV, human immunodeficiency virus; OR, odds ratio.

^a^ Not estimable in the adjusted model due to quasi‐separation; crude estimate shown.

Among analysed participants, 295/1601 (18.5%) had at least significant fibrosis (≥ 7 kPa) and 97/1601 (6.1%) had cirrhosis (> 12.5 kPa). In multivariable logistic regression adjusted for age, sex, HIV status, use of HBV antiviral therapy, and country, mild (AOR, 0.93; 95% CI, 0.60–1.43) and moderate steatosis (AOR, 1.05; 95% CI, 0.58–1.88) were not associated with fibrosis, whereas severe steatosis was associated with 58% increased odds of fibrosis (AOR, 1.58; 95% CI, 1.13–2.20). Compared to no steatosis, severe steatosis was associated with an 8‐percentage point higher absolute probability of fibrosis (average marginal effects: +8.4%, 95% CI, +19 to +14.9; Table 3; Figure 2).

**TABLE 3:** Association of degree of steatosis with hepatic fibrosis based on transient elastography.

Steatosis grade	*n* (%) with fibrosis	Crude odds ratio (95% CI)	*p*	Adjusted odds ratio (95% CI)^a^	*p*	Average marginal effects (95% CI)	*p*
S0 (none)	266/1180 (22.5%)	References		References		References	
S1 (mild)	31/139 (22.3%)	0.99 (0.65–1.50)	0.949	0.90 (0.58–1.39)	0.624	−1.79 (−8.78–5.21)	0.617
S2 (moderate)	17/63 (27.0%)	1.27 (0.72–2.25)	0.414	1.05 (0.58–1.90)	0.881	0.77 (−9.41–10.96)	0.882
S3 (severe)	78/219 (35.6%)	1.90 (1.40–2.59)	< 0.001	1.58 (1.13–2.20)	0.007	8.42 (1.90–14.94)	0.011

^a^ Adjusted for age (continuous), sex, HIV status, HBV treatment, and country.

**FIGURE 2:**
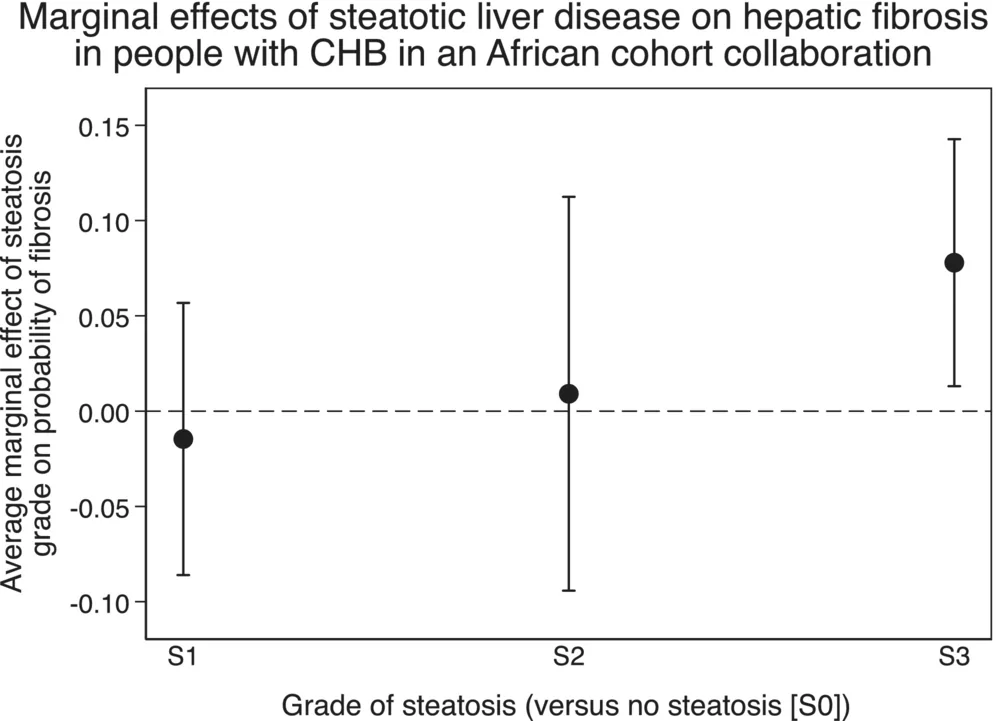
Point estimate and 95% confidence intervals around the marginal effects of increasing stage of steatosis (versus no steatosis) on the probability of hepatic fibrosis.

In exploratory analysis, over a median of 2.69 years of follow‐up after steatosis assessment, 38 participants died, including 14 from liver‐related causes (i.e., cirrhosis and/or HCC). The incidence of all‐cause mortality was 2.0% at 2 years and 3.2% at 5 years. In survival analysis, steatosis was not associated with increased all‐cause or liver‐related mortality (Tables S2 and S3).

For the 10 valid CAP measures, the IQR was documented in just under half (*n* = 754). Of these, 88.5% (667 of 754) had IQR/median of < = 0.3. In a sensitivity analysis, where patients with CAP IQR/median > 0.3 were excluded, there was no meaningful change to the results of the above analyses of associations with steatosis and fibrosis (Table S4).

At the time of CAP, 1019 (63.6%) participants also underwent B‐mode ultrasound. The overall discriminatory ability of B‐mode ultrasound was poor for any steatosis (CAP ≥ 248 dB/m), with AUROC of 0.62 (95% CI, 0.59–0.65), sensitivity of 27.8% and specificity of 95.8%. For severe steatosis (CAP ≥ 280 dB/m), performance was better but remained suboptimal, with AUROC of 0.70 (95% CI, 0.65–0.74), sensitivity of 44.0% and specificity of 95.0% (Table 4).

**TABLE 4:** Performance of B‐mode ultrasound in the diagnosis of steatosis among participants with HBV infection.

	Any grade of steatosis (CAP ≥ 248 dB/m)	Severe steatosis (CAP ≥ 280 dB/m)
AUROC	0.62 (0.59–0.64)	0.70 (0.66–0.73)
Sensitivity	28.1% (23.2–33.4)	44.5% (36.3–53.0)
Specificity	95.6% (94.0–96.9)	94.9% (93.3–96.3)
Positive predictive value	70.6% (61.9–78.4)	58.6% (48.8–67.8)
Negative predictive value	78.1% (75.4–80.6)	91.4% (89.5–93.1)

Abbreviations: AUROC, area under the received operating curve; CAP, controlled attenuation parameter.

## 4 Discussion

In this multicentre cohort study from 6 countries in Africa, a quarter of people living with CHB had comorbid SLD while 14% had severe steatosis. SLD was associated with higher education and body weight, which are rapidly increasing in the Africa region [5, 12]. Moreover, severe steatosis was associated with significant liver fibrosis (~25% of participants), but not short‐term mortality in a limited analysis over a median of 2.8 years of follow‐up. B‐mode ultrasound had suboptimal sensitivity in detecting steatosis based on TE CAP, although specificity was 95%. These data support the need to screen for metabolic comorbidities as part of HBV care in Africa. As TE with CAP is rarely available and ultrasound performed poorly, there is strong need for scalable strategies to identify people with HBV and metabolic dysfunction.

In this study, we observed a link between comorbid severe steatosis, present in 14% of the sample, and liver fibrosis in participants with CHB, building on other studies. In a meta‐analysis of studies mainly from Asia but also from Europe, Middle East, and the Americas, the pooled prevalence of any degree of steatosis among people with CHB was 32.8% [13] which is close to the 26% seen in this study. In that same study, any versus no steatosis was not associated with significant fibrosis. In a large cohort study from Taiwan, 75% of people with SLD in the context of CHB also met criteria for MASLD, and MASLD was associated with 80% higher all‐cause and 20% higher liver‐related mortality [14]. Due to the lack of data on the prevalence of metabolic parameters such as lipids, waist circumference, and blood pressure, we were unable to diagnose MASLD using international criteria in this study [2] but we observed 32% obesity and 8% diabetes in people with SLD, suggesting a high prevalence of MASLD in that group. Over an average of 3 years of follow‐up, we assessed but did not observe any difference in mortality, after adjustment for fibrosis, with or without SLD. An analysis with longer term follow‐up is warranted since there remains controversy about whether steatosis should be a criterion for HBV treatment, whether it affects the natural history of HBV‐associated liver disease, and whether antiviral treatment can alter this trajectory. Interestingly, SLD without other metabolic risk factors (diabetes, hypertension, dyslipidemia, etc.) has been associated with reduced all‐cause mortality among people with CHB [14] underscoring the need for further molecular and epidemiological research into this complex interplay.

In this study we also identified patient profiles in Africa that carry higher prevalence of hepatic steatosis. Men with CHB had more steatosis than women, which was surprising as in Africa, urban women tend to have more obesity and sedentary behaviours than men [15]. We speculate that this could be due to an increased rate of alcohol consumption, which can drive hepatic steatosis. We also identified trends, albeit not statistically significant, toward more steatosis with higher education level, which in Africa may be linked to higher socioeconomic status, obesity, and sedentary lifestyles, in addition to access to western‐style diets.

Our analysis uniquely captured a subgroup of participants with HBV/HIV coinfection, and we were surprised to observe that coinfection was associated with significantly lower odds of steatosis, after adjustment for demographic factors and body mass index. People with HIV globally are reported to have a high burden of SLD because of the virus, antiretroviral drugs, and lifestyle risk factors [9]. However, there have been few direct comparisons of SLD in people with and without HIV. In one such comparison in the U.S., the prevalence of steatosis was similar with and without HIV [16]. In a study from Zambia, steatosis was less prevalent in adults with HIV compared to HIV‐negative controls [17]. One potential explanation for our finding is that people living with HIV in sub‐Saharan Africa tend to have lower adiposity and reduced metabolic risk profiles, driven by HIV‐associated weight loss, reduced appetite, and higher rates of food insecurity—factors that may counteract the steatogenic effects of the virus and antiretroviral therapy [18]. While 9% of participants with CHB were underweight in this study, reflecting the realities of Africa, this was 23% among participants with HBV/HIV. Additionally, the specific antiretroviral regimens used in Zambia, which have shifted toward integrase inhibitor‐based regimens, may have a more favourable hepatic metabolic profile compared to older regimens historically associated with hepatic steatosis.

To shed light on alternatives to CAP for diagnosis of steatosis, we analysed B‐mode ultrasound but observed limited performance across HEPSANET sites. Our data on ultrasound for the diagnosis of steatosis are in some conflict with other results. In a meta‐analysis, ultrasound had 85% sensitivity compared to histology report of at least 20% steatosis (i.e., equivalent to S2‐S3) [19]. Among people with CHB in Taiwan, when compared to CAP, ultrasound performed by a hepatologist had AUROC of 0.79 [20]. In Korea, a strong correlation between ultrasound and CAP was also reported [21]. Beyond just detecting steatosis, in the hands of experts, B‐mode ultrasound was also able to accurately grade the level of steatosis compared to biopsy [22]. Reduced performance of ultrasound in HEPSANET compared to other published studies has several explanations. Steatosis may be underreported by radiographers in Africa, where it is not a widely recognized clinical problem and/or because they prioritized reporting of other findings due to high workloads. We noted that 15% of ultrasound reports compiled for HEPSANET did not include comment on the presence of absence of steatosis. In addition, most abdominal ultrasounds in HEPSANET were performed in a general radiology unit, while published studies were carried out in hepatology centres with liver‐focused imaging and where the study hypothesis focused on steatosis. That accuracy can be high in the hands of skilled and focused operators supports efforts to build liver‐related ultrasound capacity in Africa, including point‐of‐care, at sites providing HBV care, especially as ultrasound is a widely available and more affordable technology than TE CAP. There are also efforts underway to enhance the performance of liver ultrasound with artificial intelligence.

These data have several implications for the field. First, they provide rationale for emerging HBV care and treatment programs in Africa to integrate metabolic screening. HIV clinics in Africa increasingly assess for metabolic syndrome on a regular basis given data on accelerated cardiometabolic complications in that population. A similar focus may be needed for people with CHB. In our data, 77% of participants with CHB and SLD by TE CAP were not taking antiviral therapy, suggesting need to disseminate the 2024 WHO guidelines around management of HBV‐SLD comorbidity [6]. While we observed a link between severe steatosis and liver fibrosis, this finding was based on cross‐sectional analysis and we did not observe a mortality difference with versus without SLD, after adjusting for fibrosis; however, person‐time was limited and the total number of liver‐related deaths was small limiting statistical power. Data from this analysis also point to the need for lifestyle and pharmacological interventions for SLD at HBV clinics. Among a poll of HEPSANET sites, none had in‐house dieticians or physical activity programs (personal communication).

The major strength of this paper was our use of a non‐invasive and well‐validated imaging technique to evaluate liver steatosis across 8 clinical sites in 6 African countries, where there were limited existing published data. Another strength was our inclusion of people with HBV/HIV, who may have altered metabolism. The analysis also had weaknesses including selection bias where participants we analysed may not be representative of the overall HEPSANET sample and of all people with CHB in Africa, potentially over‐representing centres and patients with greater resources and with strong bias toward urban tertiary centres where risk factors for metabolic syndrome may be greater. Also, people with overweight/obesity and older age were more likely to have CAP data, which created potential selection bias. Our focus on populations receiving clinical care may over‐estimate population‐level prevalence of SLD due to referral of patients with features of liver disease, in addition to patients diagnosed through asymptomatic screening. Another limitation was that HEPSANET data did not capture comprehensive cardiometabolic risk factors and conditions such as lipids and waist circumference, nor alcohol use data. The network is planning to begin a prospective assessment of these comorbidities and risk factors including lifestyle characteristics that may influence liver disease outcomes. Finally, only two sites provided most people with HBV/HIV; therefore, the lower prevalence of steatosis in that group may also reflect site‐level factors and may not be sufficiently representative of all regions of Africa.

In summary, comorbid SLD was found to be both prevalent and associated with elevated liver stiffness in a cross‐sectional analysis of adults with CHB across 6 countries in Africa. Additional research in Africa is needed on the intersection of emerging cardiometabolic conditions and CHB.

## Funding

This study was supported by grants from European Association for the Study of the Liver (EASL Registry grant) and the John C. Martin Foundation. Freeman W. Chabala was supported by a training grant from the U.S. National Institutes of Health (D43TW012274). Michael J. Vinikoor is supported by grants R37AI179640 and R01AI195435 and Alana T. Brennan is supported by grant K01DK116929 from the U.S. National Institutes of Health. Philippa C. Matthews is supported by the Francis Crick Institute (funding from the UK Medical Research Council, Cancer Research UK and Wellcome; ref. CC2223), and the NIHR University College London Biomedical Research Centre.

## Conflicts of Interest

Mohamed El‐Kassas reports having served as an investigator, speaker, and/or advisory board member for AstraZeneca, Roche, MSD, AbbVie, Eva, Multicare, Mash Premier, Takeda, Organon, AUG, Inspire, HSO, Gilead, Janssen, Intercept, Rameda, FPI, Ipsen, Onxeo, Sanofi, Sandoz, Al Andalus Pharma, Minapharm, Pharco, Zeta, Alfa Cure, Bayer, Oncoustics, PDC, Acino, Opella, Spimaco, and Novo Nordisk. The remaining authors report no conflicts of interest.

## Supporting information


**Table S1:** Correlates of evaluation for steatosis with TE CAP.
**Table S2:** Predictors of all‐cause mortality.
**Table S3:** Predictors of liver‐related mortality.
**Table S4:** Sensitivity analysis of factors associated with SLD among people with CHB at selected HEPSANET sites when CAP median/IQR was < 0.3.
**Figure S1:** Confounders (among variables available) in the association of grade of steatosis with hepatic fibrosis were identified using a directed acyclic graph and these were included in multivariable models.

## Data Availability

Data from the HEPSANET study are available for secondary use following an application and review by the HEPSANET steering committee and site leaders, through submission of a data analysis application request (please consult www.hepsanet.org/data).

## About HEPSANET

The Hepatitis B in Africa Collaborative Network (HEPSANET) is a cohort collaboration focused on generating and disseminating data to accelerate elimination of hepatitis B in the Africa region. Currently HEPSANET has 15 cohorts involved across 12 countries in Africa. Each cohort features a clinical site that provides hepatitis B care and each cohort has a local principal investigator (PI) that serves as the focal point for the network. The network also has a steering committee of researchers focused on hepatitis B in Africa.

Below are names of the cohorts with current site PIs: Alice Guingane (Bogodogo University Hospital Center, Ouagadougou, Burkina Faso), Roger Sombie (Service d'Hépato‐Gastro‐Entérologie, Centre Hospitalier Universitaire Yalgado Ouédraogo, Ouagadougou, Burkina Faso), Asgeir Johannessen (St Paul's Hospital Millenium Medical College, Addis Ababa, Ethiopia), Edith Okeke (Jos University Teaching Hospital, University of Jos, Jos, Nigeria), Mohamed El‐Kassas (Endemic Medicine Department, Capital University, Cairo, Egypt), Gilles Wandeler (SEN‐B cohort, Service de Maladies Infectieuses et Tropicales, Fann University Hospital, Dakar, Senegal), Ibrahima Diallo (Department of Gastroenterology and Hepatology, Hôpital Principal de Dakar, Dakar, Senegal), Joan Rugemalila (Department of Medicine, Muhimbili National Hospital, Dar Es Salaam, Tanzania), Wendy Spearman (Division of Hepatology, Department of Medicine, Groote Schuur Hospital, University of Cape Town, Cape Town, South Africa), Hans Prozesky (Division of Infectious Disease, Department of Medicine, Tygerberg Hospital, University of Stellenbosch, Stellenbosch, South Africa), Alex Stockdale (Malawi Epidemiology and Intervention Research Unit, Chilumba, Karonga District, Malawi), Maud Lemoine (PROLIFICA cohort, Medical Research Council Gambia at the London School of Tropical Medicine, Fajara, Gambia), Babatunde Duduyemi (University of Sierra Leone Teaching Hospitals Complex, Freetown, Sierra Leone), Mary Afihene (Kumasi cohort, Komfo Anokye Teaching Hospital, Kwame Nkrumah University of Science and Technology, Kumasi, Ghana), Edford Sinkala (Hep‐Mec cohort, Lusaka, Zambia).

Below are the names and affiliations of the current HEPSANET steering committee: Asgeir Johannessen (Institute of Clinical Medicine, University of Oslo, Oslo, Norway; St Paul's Hospital Millenium Medical College, Addis Ababa, Ethiopia), Roger Sombie (Centre Hospitalier Universitaire Yalgado Ouédraogo, Ouagadougou, Burkina Faso), Alex Stockdale (Malawi Liverpool Wellcome Research Programme, Blantyre, Malawi; Department of Clinical Infection, Microbiology and Immunology, University of Liverpool, Liverpool, United Kingdom), Alice Guingane (Bogodogo University Hospital Center, Ouagadougou, Burkina Faso), Michael Vinikoor (Division of Infectious Disease, University of Alabama at Birmingham, Birmingham, Alabama, USA; Centre for Infectious Disease Research in Zambia), Edith Okeke (Department of Medicine, University of Jos, Jos, Nigeria), Maud Lemoine (Division of Digestive Diseases, Hepatology Section, Department of Metabolism, Digestion and Reproduction, Imperial College London, London, United Kingdom; Medical Research Council Gambia at the London School of Tropical Medicine, Fajara, Gambia), Philippa Matthews (Francis Crick Institute, London, United Kingdom; Division of Infection and Immunity, University College London, London, UK), Wendy Spearman (Division of Hepatology, Department of Medicine, University of Cape Town, Cape Town, South Africa), Adria Ramirez (Department of Infectious Diseases, Bern University Hospital, University of Bern, Bern, Switzerland; Department of Infectious and Tropical Diseases, Fann University Hospital, Dakar, Senegal).
