# Supplementary material for: Hepatic Steatosis in People With Chronic Hepatitis B in Africa: Prevalence, Correlates, and Clinical Significance
Source: J Viral Hepat. 2026 Oct 4;33(11):e70241. doi: 10.1111/jvh.70241 (PMC13635601; doi:10.1111/jvh.70241)
Supplement: Supplementary file 1 [file JVH-33-0-s001.docx]

**Supplementary figures and tables**

Supplementary Figure 1 caption.

Confounders (among variables available) in the association of grade of steatosis with hepatic fibrosis were identified using a directed acyclic graph and these were included in multivariable models.

| **Supplementary Table 1. Correlates of evaluation for steatosis with TE CAP** | | |
| --- | --- | --- |
|  | **Crude odds ratio (95% CI)** | **Adjusted odds ratio (95% CI)** |
| Age, years |  |  |
| 12-29 | Reference | Reference |
| 30-39 | 1.58 (1.36–1.83) | 1.51 (1.15–1.97) |
| 40-49 | 1.72 (1.46–2.03) | 1.76 (1.26–2.46) |
| 50+ | 1.97 (1.63–2.39) | 1.27 (0.86–1.87) |
| Sex |  |  |
| Female | Reference | Reference |
| Male | 0.95 (0.84–1.07) | 1.19 (0.96–1.47) |
| Marital status |  |  |
| Single | Reference | Reference |
| Married/cohabiting | 1.37 (1.20–1.57) | 1.19 (0.92–1.53) |
| Divorced/widowed | 1.18 (0.88–1.60) | 1.63 (0.72–3.71) |
| Education |  |  |
| None/primary | Reference | Reference |
| Secondary | 1.01 (0.83–1.22) | 1.48 (1.08–2.02) |
| Tertiary and above | 0.83 (0.69–0.99) | 1.34 (1.01–1.78) |
| Body mass index |  |  |
| <18.5 | 1.13 (0.90–1.43) | 1.33 (0.87–2.02) |
| 18.5-24.9 | Reference | Reference |
| 25-29.9 | 1.71 (1.46–2.01) | 1.57 (1.23–2.00) |
| >=30 | 1.98 (1.61–2.42) | 1.70 (1.23–2.33) |
| Diabetes history |  |  |
| No | Reference | Reference |
| Yes | 1.51 (1.07–2.14) | 0.97 (0.53–1.76) |
| HIV status |  |  |
| Negative | Reference | Reference |
| Positive | 1.08 (0.93–1.24) | 7.82 (4.02–15.21) |
| Treatment status |  |  |
| Not on antivirals | Reference | Reference |
| On antivirals | 0.70 (0.63–0.79) | 0.60 (0.47–0.76) |
| Year of enrollment |  |  |
| <2010 | Reference | Reference |
| 2010-15 | 1.72 (1.30–2.27) | 1.75 (1.21–2.54) |
| 2015-20 | 2.87 (2.23–3.69) | 6.55 (4.59–9.37) |
| >=2020 | 3.32 (2.59–4.25) | 7.25 (5.10–10.30) |
| Estimates were based on logistic regression.  Abbreviations: TE, transient elastography; CAP, controlled attenuation parameter; CI, confidence intervals | | |

| **Supplementary Table 2. Predictors of all-cause mortality** | | | | |
| --- | --- | --- | --- | --- |
|  | **Crude HR**  **(95% CI)** | **p** | **Adjusted HR**  **(95% CI)** | **p** |
| Steatosis grade |  |  |  |  |
| S0 (none) | Reference |  | Reference |  |
| S1 (mild) | 1.02 (0.36-2.90) | 0.973 | 2.39 (0.77-7.42) | 0.132 |
| S2 (moderate) | 1.20 (0.29-5.02) | 0.805 | 1.76 (0.40-7.77) | 0.456 |
| S3 (severe) | 0.50 (0.15-1.63) | 0.250 | 0.65 (0.14-2.96) | 0.578 |
| Age, years |  |  |  |  |
| 12-29 | Reference |  | Reference |  |
| 30-39 | 0.79 (0.31-2.05) | 0.631 | 0.37 (0.12-1.13) | 0.080 |
| 40-49 | 1.85 (0.77-4.47) | 0.171 | 0.57 (0.20-1.66) | 0.305 |
| 50+ | 1.85 (0.70-4.94) | 0.218 | 0.78 (0.26-2.40) | 0.670 |
| Sex |  |  |  |  |
| Female | Reference |  | Reference |  |
| Male | 1.51 (0.76-3.00) | 0.235 | 1.30 (0.62-2.73) | 0.479 |
| Diabetes history |  |  |  |  |
| No | Reference |  | Reference |  |
| Yes | 0.85 (0.12-6.23) | 0.875 | 0.63 (0.08-4.77) | 0.654 |
| HIV status |  |  |  |  |
| Negative | Reference |  | Reference |  |
| Positive | 8.34 (4.17-16.69) | <0.001 | 9.28 (3.17-27.18) | <0.001 |
| Median LSM |  |  |  |  |
| <7 kPa | Reference |  | Reference |  |
| 7-12.5 kPa | 1.08 (0.40-2.91) | 0.880 | 0.96 (0.32-2.91) | 0.940 |
| >12.5 kPa | 12.53 (6.30-24.92) | <0.001 | 10.25 (4.71-22.26) | <0.001 |
| Treatment status |  |  |  |  |
| Not on antivirals | Reference |  | Reference |  |
| On antivirals | 5.09 (2.46-10.53) | <0.001 | 1.27 (0.42-3.80) | 0.672 |
| Estimates were based on Cox proportional hazards regression.  Abbreviations: HR, hazard ratio; LSM, liver stiffness measurement | | | | |

| **Supplementary Table 3. Predictors of liver-related mortality** | | | | |
| --- | --- | --- | --- | --- |
|  | **Crude HR**  **(95% CI)** | **p** | **Adjusted HR**  **(95% CI)** | **p** |
| Steatosis grade |  |  |  |  |
| S0 (none) | Reference |  | Reference |  |
| S1 (mild) | 2.27 (0.62-8.26) | 0.213 | 7.70 (1.60-37.01) | 0.011 |
| S2 (moderate) | 1.75 (0.22-13.72) | 0.592 | 2.58 (0.30-22.48) | 0.392 |
| S3 (severe) | 0.00 (0.00-.) | 1.000 | 0.00 (0.00-.) | 1.000 |
| Age, years |  |  |  |  |
| 12-29 | Reference |  | Reference |  |
| 30-39 | 0.70 (0.14-3.46) | 0.660 | 0.25 (0.04-1.56) | 0.138 |
| 40-49 | 2.24 (0.56-8.96) | 0.255 | 0.38 (0.07-2.08) | 0.264 |
| 50+ | 1.20 (0.20-7.21) | 0.839 | 0.34 (0.05-2.40) | 0.281 |
| Sex |  |  |  |  |
| Female | Reference |  | Reference |  |
| Male | 1.23 (0.41-3.68) | 0.707 | 0.76 (0.23-2.56) | 0.660 |
| Diabetes history |  |  |  |  |
| No | Reference |  | Reference |  |
| Yes | 0.00 (0.00-.) | 1.000 | 0.00 (0.00-.) | 1.000 |
| HIV status |  |  |  |  |
| Negative | Reference |  | Reference |  |
| Positive | 4.83 (1.62-14.41) | 0.005 | 3.70 (0.86-15.83) | 0.078 |
| Median LSM |  |  |  |  |
| <7 kPa | Reference |  | Reference |  |
| 7-12.5 kPa | 0.97 (0.11-8.68) | 0.978 | 1.34 (0.14-12.50) | 0.796 |
| >12.5 kPa | 30.96 (9.51-100.77) | <0.001 | 34.04 (8.45-137.23) | <0.001 |
| Treatment status |  |  |  |  |
| Not on antivirals | Reference |  | Reference |  |
| On antivirals | 6.51 (1.80-23.50) | 0.004 | 3.37 (0.73-15.62) | 0.121 |
| Estimates were based on Cox proportional hazards regression.  Abbreviations: HR, hazard ratio; LSM, liver stiffness measurement | | | | |

| **Supplementary Table 4. Sensitivity analysis of factors associated with SLD among people with CHB at selected HEPSANET sites when CAP median/IQR was <0.3** | | |
| --- | --- | --- |
|  | **Crude odds ratio**  **(95% CI)** | **Adjusted odds ratio**  **(95% CI)** |
| Age, years |  |  |
| 12-29 | Reference | Reference |
| 30-39 | 2.49 (1.56-3.97) | 1.72 (0.89-3.33) |
| 40-49 | 4.30 (2.56-7.24) | 2.67 (1.24-5.76) |
| 50+ | 6.50 (3.42-12.33) | 2.47 (0.97-6.26) |
| Sex |  |  |
| Female | Reference | Reference |
| Male | 1.31 (0.93-1.84) | 1.80 (1.09-2.99) |
| Marital status |  |  |
| Single | Reference | Reference |
| Married/cohabiting | 1.68 (1.09-2.58) | 0.75 (0.40-1.42) |
| Divorced/widowed | 0.41 (0.12-1.45) | 1.64 (0.17-15.94) |
| Education |  |  |
| None/primary | Reference | Reference |
| Secondary | 1.25 (0.65-2.40) | 0.89 (0.38-2.12) |
| Tertiary and above | 1.96 (1.19-3.24) | 1.02 (0.51-2.06) |
| Body mass index |  |  |
| <18.5 | 0.28 (0.08-0.92) | 0.25 (0.03-1.96) |
| 18.5-24.9 | Reference | Reference |
| 25-29.9 | 3.97 (2.61-6.04) | 5.02 (2.92-8.65) |
| >=30 | 7.87 (4.58-13.53) | 9.09 (4.35-19.02) |
| Diabetes history |  |  |
| No | Reference | Reference |
| Yes | 1.73 (0.46-6.50) | 0.29 (0.02-3.48) |
| HIV status |  |  |
| Negative | Reference | Reference |
| Positive | 0.36 (0.19-0.69) | 1.00 (1.00-1.00) |
| Treatment status |  |  |
| Not on antivirals | Reference | Reference |
| On antivirals | 0.74 (0.52-1.07) | 1.33 (0.76-2.34) |
| Estimates were based on logistic regression.  Abbreviations: CHB, chronic hepatitis B; SLD, steatotic liver disease; CAP, controlled attenuation parameter; CI, confidence intervals | | |
